# Longer sleep is associated with lower BMI and favorable metabolic profiles in UK adults: Findings from the National Diet and Nutrition Survey

**DOI:** 10.1371/journal.pone.0182195

**Published:** 2017-07-27

**Authors:** Gregory D. M. Potter, Janet E. Cade, Laura J. Hardie

**Affiliations:** 1 Division of Epidemiology and Biostatistics, School of Medicine, University of Leeds, Leeds, West Yorkshire, England; 2 Nutritional Epidemiology Group, University of Leeds, Leeds, West Yorkshire, England; Medizinische Universitat Innsbruck, AUSTRIA

## Abstract

Ever more evidence associates short sleep with increased risk of metabolic diseases such as obesity, which may be related to a predisposition to non-homeostatic eating. Few studies have concurrently determined associations between sleep duration and objective measures of metabolic health as well as sleep duration and diet, however. We therefore analyzed associations between sleep duration, diet and metabolic health markers in UK adults, assessing associations between sleep duration and 1) adiposity, 2) selected metabolic health markers and 3) diet, using National Diet and Nutrition Survey data. Adults (n = 1,615, age 19–65 years, 57.1% female) completed questions about sleep duration and 3 to 4 days of food diaries. Blood pressure and waist circumference were recorded. Fasting blood lipids, glucose, glycated haemoglobin (HbA1c), thyroid hormones, and high-sensitivity C-reactive protein (CRP) were measured in a subset of participants. We used regression analyses to explore associations between sleep duration and outcomes. After adjustment for age, ethnicity, sex, smoking, and socioeconomic status, sleep duration was negatively associated with body mass index (-0.46 kg/m^2^ per hour, 95% CI -0.69 to -0.24 kg/m^2^, p < 0.001) and waist circumference (-0.9 cm per hour, 95% CI -1.5 to -0.3cm, p = 0.004), and positively associated with high-density lipoprotein cholesterol (0.03 mmol/L per hour, 95% CI 0.00 to 0.05, p = 0.03). Sleep duration tended to be positively associated with free thyroxine levels and negatively associated with HbA1c and CRP (p = 0.09 to 0.10). Contrary to our hypothesis, sleep duration was not associated with any dietary measures (p ≥ 0.14). Together, our findings show that short-sleeping UK adults are more likely to have obesity, a disease with many comorbidities.

## Introduction

Including undiagnosed cases, approximately 4.5 million people in the UK have diabetes, and it was estimated that in 2015 about 415 million 20–70 year old adults had diabetes worldwide [1]. Roughly 24,000 individuals die prematurely each year in the UK as result of diabetes [2]. Diabetes therefore is a large economic burden, costing the National Health Service in the UK about £10 billion in direct costs each year, 10% of its budget [3]. Type 2 diabetes accounts for the majority of diabetes cases and costs, and obesity is the most potent risk factor for type 2 diabetes. Although not all people with obesity develop the disease, obesity accounts for much of type 2 diabetes risk [4]. About 59% of women and 68% of men in the UK are now overweight or have obesity [5]. Obesity predisposes the affected to metabolic dysfunction, and central obesity appears to explain much of this [6]. Metabolic syndrome (comprising central obesity, dyslipidemia, hyperglycemia, and hypertension) is a cluster of risk factors that also increases risk of type 2 diabetes [7] and is thought to affect about a quarter of adults worldwide [8]. Identifying the lifestyle factors that influence risk of obesity, metabolic syndrome, and type 2 diabetes is therefore a public health priority.

Short sleep is increasingly common in many countries, and findings from an analysis of ~ 250,000 sleep questionnaires worldwide suggest that sleep duration on workdays has declined by ~ 37 minutes in the last decade [9]. Large epidemiologic studies have consistently linked short sleep to type 2 diabetes, (central) obesity and metabolic syndrome [10–13], and some of the mechanisms contributing to associations between short sleep and metabolic diseases are beginning to be understood [14]. Among these mechanisms, short sleep may affect dietary choices, predisposing individuals to selection of energy-dense, rewarding foods and non-homeostatic eating [15]. The increases in type 2 diabetes, obesity and metabolic syndrome prevalence that have occurred concurrently with declines in sleep duration are hence unlikely to be mere coincidences.

We therefore used data from years 1–4 of the National Diet and Nutrition Survey Rolling Programme (NDNS-RP) to determine whether sleep duration was associated with diet, adiposity, glucose and lipid metabolism, metabolic syndrome criteria, thyroid function, and inflammation in UK adults. In doing so, we are the first to concurrently report on associations between sleep duration and nutrient intakes, and sleep duration and objective measures of metabolic health in UK adults, to our knowledge. We hypothesized that short sleep would be associated with 1) less healthy dietary habits, 2) obesity, 3) dysglycemia, 4) dyslipidemia, 5) metabolic syndrome, 6) impaired thyroid function, and 7) higher systemic inflammation.

## Materials and methods

The NDNS-RP aims to track diet and nutritional status in 1,000 individuals per year (500 children aged 1.5–18 years, and 500 adults aged 19 years and over) living in private households in England, Northern Ireland, Scotland, and Wales. NDNS-RP results are used to monitor diet trends in the UK to develop policies to improve health, and data are available online at the UK Data Service website [16]. A detailed overview of the NDNS-RP methods has been described elsewhere [17].

Briefly, households were randomly selected from the Postcode Address File (all addresses in the UK) and grouped into units by location. Information about the purpose of the NDNS-RP was then sent to addresses randomly selected from these units, after which interviewers contacted the households to arrange visits to recruit participants and distribute diet diaries for 4 consecutive days of diet recording. With help from an interviewer, participants completed a computer-assisted personal interview to collect data on background and lifestyle. Height and weight were measured at these visits also. Individuals who completed diet diaries for at least 3 of the 4 days they were asked to record after the interviews were eligible for visits by nurses for further anthropometry and physiological measures. The Oxfordshire A Research Ethics Committee approved the study, which was conducted in accordance with the Declaration of Helsinki. We used the data for 19–65 year old, non-pregnant adults. Written informed consent was obtained from all participants. Participants who provided blood samples were compensated with £15 in high street shop vouchers for their contributions.

### Sleep

Participants were asked the following 2 computer-assisted personal interview questions about habitual sleep duration at participant interviews: “How long (do you) usually sleep on week nights?” and “How long (do you) usually sleep on weekend nights?” We therefore estimated participants’ mean daily sleep duration using the formula ((5 x self-reported usual weekday sleep duration) + (2 x self-reported usual weekend sleep duration) / 7)). Participants were not asked about napping or shift work schedules.

### Blood pressure

After resting for 5 minutes in a seated position, blood pressure was measured 3 times with 1 minute between readings, using an automated sphygmomanometer (Omron HEM907, Kyoto, Japan). To avoid behaviours that can acutely influence blood pressure, participants had not eaten, exercised, drunk alcohol, or smoked in the preceding 30 minutes. For consistency, we used the mean of the second and third readings because the first reading is often the highest [18].

### Anthropometry

Participants were measured for height and weight using portable stadiometers and weighing scales, respectively. Nurses measured waist circumference using tape measures at follow-up household visits. Height, weight, and waist circumference were measured twice. If there were unacceptable discrepancies (height ± 0.5 cm, weight ± 0.2 kg, waist circumference, ± 3 cm) then a third measurement was completed, and the mean value of the most similar 2 measurements was used.

### Blood measures

After anthropometry and blood pressure measures were taken, eligible participants provided up to approximately 35 ml of fasted blood via venepuncture. Venepuncture exclusion criteria included bleeding and clotting disorders, use of anticoagulant medications, an epileptic fit within the previous 5 years, and self-disclosed infection with hepatitis B or human immunodeficiency virus. To stabilise samples, blood was collected into tubes containing appropriate anticoagulants/stabilising agents. Samples were then processed at suitably equipped field laboratories located within 2 hours and stored at -20 to -80°C before subsequent analyses after transportation to the National Health Service laboratory at Addenbrooke’s Hospital or the Human Nutrition Research centre in Cambridge, UK. Detailed information about sample processing [19] and the range of analytes measured [20] is available online.

As we were interested in the associations between sleep duration and metabolic health, we analysed data for analytes of particular clinical relevance to metabolic diseases such as type 2 diabetes and obesity. Specifically, we used data for the following analytes: 1) fasting glucose and glycated haemoglobin (HbA1c) (measures of glucose metabolism), 2) high-density lipoprotein (HDL) cholesterol, low-density lipoprotein (LDL) cholesterol, and triglycerides (measures of lipid metabolism), 3) free triiodothyronine (T3), free thyroxine (T4), and thyroid-stimulating hormone (TSH) (measures of thyroid function), and 4) high-sensitivity C-reactive protein (CRP) (a measure of systemic inflammation).

### Metabolic syndrome

We determined whether participants had the metabolic syndrome using the International Diabetes Federation (2006) criteria. According to these criteria, metabolic syndrome is defined as central obesity (using waist circumference values that are ethnicity- and sex-specific) plus at least two of the following: raised blood pressure (systolic blood pressure ≥ 130 mmHg, diastolic blood pressure ≥ 85 mmHg, or treatment for hypertension), raised fasting plasma glucose (≥ 5.6 mmol/L or treatment for diabetes), raised triglycerides (≥ 1.7 mmol/L or treatment for hypertriglyceridemia), and reduced HDL cholesterol (< 40 mg/dL for males, < 50 mg/dL for females, or treatment for low HDL cholesterol levels). In accordance with the criteria, central obesity was assumed if BMI was > 30 kg/m^2^ [8].

### Diet

Participants completed 3 to 4 food diaries on consecutive days. These were collected no later than 3 days after the final diet day. Participants were asked to provide detailed descriptions of all items consumed, including time and estimated (not weighed) quantity of consumption. Weekend days were over-represented in year 1 of the NDNS-RP and were subsequently under-sampled in year 2 to address this.

Food diary data were processed by trained coders and editors who entered diaries into the Medical Research Council Human Nutrition Research dietary assessment system, Diet In Nutrients Out, using food composition data for >6,000 foods [21]. Component parts of composite items (such as sandwiches) were assigned individual food codes. Detailed information on data coding and editing is provided in Appendix A of the NDNS-RP official report [20].

We decided to assess energy intake; macronutrient intakes (including alcohol); fibre, fruit (excluding fruit juice) and vegetable intakes as markers of healthy dietary habits. We also assessed non-milk extrinsic sugar, saturated fatty acid, *trans*-fatty acid, and sodium intakes as indices of unhealthy dietary habits. Dietary fibre comprised non-starch polysaccharides, as defined by the Englyst method [22]. Non-milk extrinsic sugars were defined as sugars added during processing or by participants, sugars in fruit juices, and 50% of sugars in canned, dried and stewed fruits. Our choices of which foods and nutrients to analyse were partly informed by various diet quality indices [23].

### Statistics

Linear regression analyses were used to test associations between sleep duration and 1) energy intake and macronutrient intakes (including macronutrient intakes as percentages of total energy intake); 2) indices of diet quality, including fibre, saturated fatty acids, *trans*-fatty acids, total sugar, non-milk extrinsic sugar, sodium, total fruit, and total vegetable intakes; 3) body mass index (BMI); 4) waist circumference; 5) blood measures (fasting glucose, HbA1c, HDL cholesterol, LDL cholesterol, triglycerides, free T3, free T4, TSH, and CRP); and 6) metabolic syndrome score (out of the 5 criteria). We divided participants by tertiles of sleep duration to produce short, middle and long sleep categories and then used binary logistic regression analysis to determine whether metabolic syndrome prevalence differed between sleep duration categories. We used directed acyclic graphs to select variables to adjust for, and models were adjusted for age, ethnicity, sex, smoking, and socioeconomic status. Additional models included BMI as an adjustment for all outcomes other than indices of diet quality.

As meta-analysis has shown there may not be simple linear relationships between sleep duration and diabetes risk [10], we used restricted cubic splines to model relationships between sleep duration and metabolic outcomes. The splines comprised 4 polynomial segments separated by 5 knots (at the following percentiles of sleep duration: 5, 27.5, 50, 72.5, and 95, as recommended by Harrell [24]), with linear regions before the first knot and after the last.

Alcohol and CRP data were not normally distributed and so they were log transformed. We report data as means ± SDs, and data are from adjusted models, unless otherwise indicated. P values ≤ 0.05 were deemed significant. Statistical analyses were completed in Stata version 13 (Texas, USA).

## Results

NDNS-RP data from years 1–4 are available for 1,692 19 to 65 year old adults. As not all participants were willing to give blood or met the blood sample eligibility criteria, blood data are available for only 51% of all participants, and some participants do not have data for various measures because of missing or invalid measurements. After excluding 75 participants without sleep data and 2 participant participants because of pregnancy/breastfeeding, we analyzed data for the remaining 1,615 non-pregnant adults (Table 1), of whom 448 were aged 19 to 34, 655 were aged 35 to 50, and 512 were aged 51 to 65. Two participants with TSH levels more than four times higher than the next highest value were excluded from thyroid hormone analyses. In total, 24.8% of participants reported being current smokers (sleep duration 7.15 ± 1.38 hours), 20.5% ex-smokers (sleep duration 7.14 ± 1.18 hours) and 54.7% reported never having smoked regularly (sleep duration 7.24 ± 1.18 hours). Men reported sleeping 7.17 ± 1.15 hours, women 7.22 ± 1.29 h.

**Table 1:** National Diet and Nutrition Survey Rolling Programme participant (n = 1,615) characteristics, stratified by tertiles of mean sleep duration.

Characteristic	Shortest third of sleep duration (5.88 ± 0.86 hours, n = 538)	Middle third of sleep duration (7.26 ± 0.26 hours, n = 538)	Longest third of sleep duration (8.44 ± 0.66 hours, n = 539)
Age (y)	44.7 ± 12.2	43.8 ± 12.5	41.2 ± 13.2
Ethnicity (% white)	92.8	89.4	88.5
Sex (% female)	55.4	53.5	62.3
Smoking (% current smokers)	27.3	23.2	23.8
Occupation (% managerial and professional)	50	57.6	47.1
*Diet*			
Energy (kcals)	1,822 ± 606	1,885 ± 578	1,783 ± 583
Carbohydrate (g)	218 ± 73	225 ± 73	215 ± 71
Carbohydrate (% total energy)	45.6 ± 8.0	45.2 ± 7.2	45.7 ± 7.6
Fat (g)	67 ± 27	71 ± 26	66 ± 26
Fat (% total energy)	32.7 ± 6.6	33.3 ± 6.1	33.1 ± 6.5
Protein (g)	73 ± 31	76 ± 23	72 ± 24
Protein (% total energy)	16.3 ± 3.9	16.5 ± 3.5	16.6 ± 4.3
Alcohol (g)	15.5 ± 25.6	14.7 ± 21.6	13.2 ± 23.7
Fibre (g)	13.5 ± 5.1	14.3 ± 5.2	13.4 ± 5.0
*Metabolism*			
BMI (kg/m^2^)	28.6 ± 5.5 (n = 499)	27.3 ± 5.3 (n = 506)	27.1 ± 5.4 (n = 505)
Waist circumference (cm)	95 ± 15 (n = 405)	92 ± 15 (n = 403)	91 ± 15 (n = 395)
Fasting glucose (mmol/L)	5.32 ± 1.35 (n = 252)	5.26 ± 1.12 (n = 246)	5.09 ± 1.14 (n = 247)
HbA1c (%)	5.64 ± 0.70 (n = 271)	5.55 ± 0.57 (n = 260)	5.46 ± 0.55 (n = 254)
HDL cholesterol (mmol/L)	1.45 ± 0.43 (n = 272)	1.53 ± 0.41 (n = 267)	1.54 ± 0.47 (n = 257)
LDL cholesterol (mmol/L)	3.24 ± 0.95 (n = 266)	3.27 ± 1.00 (n = 262)	3.15 ± 0.96 (n = 250)
Triglycerides (mmol/L)	1.50 ± 1.14 (n = 270)	1.25 ± 0.83 (n = 267)	1.32 ± 1.05 (n = 257)
Systolic blood pressure (mmHg)	125 ± 14 (n = 309)	125 ± 16 (n = 319)	124 ± 16 (n = 298)
Diastolic blood pressure (mmHg)	75 ± 10 (n = 309)	75 ± 11 (n = 319)	75 ± 11 (n = 298)
CRP (mg/L)	3.40 ± 4.15 (n = 273)	2.79 ± 3.11 (n = 267)	3.35 ± 5.25 (n = 257)
Free T3 (pmol/L)	5.06 ± 0.58 (n = 102)	5.06 ± 0.52 (n = 100)	5.04 ± 0.51 (n = 79)
Free T4 (pmol/L)	13.07 ± 1.91 (n = 102)	13.23 ± 2.04 (n = 100)	13.39 ± 1.74 (n = 79)
TSH (mIU/L)	2.57 ± 2.18 (n = 102)	2.59 ± 1.76 (n = 100)	2.63 ± 1.68 (n = 79)

Data are means ± SDs.

Legend: BMI (body mass index), CRP (C-reactive protein), HbA1c (glycated haemoglobin), HDL (high-density lipoprotein), LDL (low-density lipoprotein), TSH (thyroid-stimulating hormone), T3 (triiodothyronine), T4 (thyroxine).

### Sleep and diet

Sleep duration was not associated with any dietary measure in the unadjusted and adjusted linear regression analyses (p ≥ 0.10, Table 2).

**Table 2:** Sleep duration and dietary intakes (n = 1,615).

	Unadjusted model		Adjusted model^a^	
Characteristic	Coefficient per additional hour of sleep (95% CI)	P value	Coefficient per additional hour of sleep (95% CI)	P value
Energy (kcals)	3 (-20 to 27)	0.78	3 (-17 to 24)	0.75
Carbohydrate (g)	1 (-2 to 3)	0.72	0 (-3 to 2)	0.83
Carbohydrate (% total energy)	0.0 (0.0 to 0.0)	0.74	-0.2 (-0.5 to 0.1)	0.13
Fat (g)	0 (-1 to 1)	0.55	0 (-1 to 1)	0.52
Fat (% total energy)	0.1 (-0.1 to 0.4)	0.27	0.2 (-0.1 to 0.4)	0.25
Protein (g)	0 (-1 to 1)	1.0	0 (-1 to 1)	0.79
Protein (% total energy)	0.0 (-0.1 to 0.2)	0.77	0.1 (-0.1 to 0.2)	0.47
Alcohol^b^	0.0 (-0.2 to 0.2)	0.90	0.0 (-0.2 to 0.2)	0.83
Fibre (g)	0.0 (-0.2 to 0.2)	0.71	0.1 (-0.1 to 0.3)	0.33
Sugar (g)	-1 (-2 to 1)	0.42	-1 (-2 to 1)	0.38
NMES (g)	0 (-1 to 1)	0.95	-1 (-2 to 1)	0.45
*Trans*-fatty acids (g)	0.00 (-0.03 to 0.02)	0.75	0.00 (-0.03 to 0.03)	0.94
Saturated fatty acids (g)	0.0 (-0.5 to 0.4)	0.88	0.0 (-0.4 to 0.5)	0.85
Sodium (g)	0.02 (0.00 to 0.06)	0.10	0.02 (0.00 to 0.05)	0.14
Fruits (not juices) (g)	-2 (-6 to 2)	0.32	0 (-4 to 4)	0.99
Vegetables (g)	1 (-3 to 5)	0.62	2 (-2 to 7)	0.28

Legend: CI (confidence interval), NMES (non-milk extrinsic sugars).

^a^Adjusted for age, ethnicity, smoking, socioeconomic status, and sex.

^b^Alcohol data were log transformed and are thus ratios.

In the models that also adjusted for BMI (n = 1,510), sleep duration was again not associated with energy (-2 kcals per additional hour of sleep, 95% CI -24 to 20 kcals, p = 0.84), carbohydrate (-1 g per additional hour of sleep, 95% CI -3 to 2 g, p = 0.66), fat (0 g per additional hour of sleep, 95% CI -1 to 1 g, p = 0.86), or protein (0 g per additional hour of sleep, 95% CI -1 to 1 g, p = 0.77) intakes.

### Sleep and metabolic health

After adjustment for age, ethnicity, sex, smoking, and socioeconomic status, HDL cholesterol was 0.03 mmol/L higher per additional hour of sleep (95% CI 0.00 to 0.05 mmol/L, p = 0.03, Table 3).

**Table 3:** Sleep duration and measures of metabolic health.

	Unadjusted model		Adjusted model^a^		
Characteristic	Coefficient per additional hour of sleep (95% CI)	P value	Coefficient per additional hour of sleep (95% CI)	P value	n
BMI (kg/m^2^)	-0.58 (-0.81 to -0.36)	< 0.001	-0.46 (-0.69 to -0.24)	< 0.001	1,510
Waist circumference (cm)	-1.4 (-.2.1 to -0.8)	< 0.001	-0.9 (-1.5 to -0.3)	0.004	1,203
HbA1c (%)	-0.05 (-0.09 to -0.01)	0.006	-0.03 (-0.07 to 0.00)	0.09	785
Fasting glucose (mmol/L)	-0.05 (-0.12 to 0.02)	0.18	-0.03 (-0.10 to 0.04)	0.44	745
HDL cholesterol (mmol/L)	0.02 (0.00 to 0.05)	0.08	0.03 (0.00 to 0.05)	0.03	795
Triglycerides (mmol/L)	-0.07 (-0.13 to -0.01)	0.02	-0.05 (-0.10 to 0.01)	0.11	794
LDL cholesterol (mmol/L)	-0.02 (-0.08 to 0.04)	0.48	0.01 (-0.05 to 0.07)	0.73	778
Free T3 (pmol/L)	0.00 (-0.06 to 0.06)	0.97	-0.01 (-0.06 to 0.04)	0.72	281
Free T4 (pmol/L)	0.18 (-0.02 to 0.38)	0.08	0.17 (-0.04 to 0.38)	0.10	281
TSH (mIU/L)	0.03 (-0.17 to 0.24)	0.74	0.03 (-0.18 to 0.23)	0.79	281
CRP^b^	-0.13 (-0.25 to 0.00)	0.05	-0.11 (-0.23 to 0.02)	0.09	797
Number of metabolic syndrome factors (out of 5^c^)	-0.10 (-0.20 to -0.02)	0.02	-0.04 (-0.13 to 0.05)	0.36	554
	Metabolic syndrome OR (95% CI)	P value	Metabolic syndrome OR (95% CI)	P value	n
Shortest third of sleep duration (5.88 ± 0.79 hours)^d^	1.33 (0.84 to 2.13)	0.23	1.22 (0.73 to 2.06)	0.44	175
Longest third of sleep duration (8.38 ± 0.65 hours)^c^	1.05 (0.65 to 1.68)	0.85	1.18 (0.69 to 2.01)	0.55	184

Legend: BMI (body mass index), CI (confidence interval), CRP (C-reactive protein), HbA1c (glycated haemoglobin), HDL (high-density lipoprotein), LDL (low-density lipoprotein), OR (odds ratio), T3 (triiodothyronine), T4 (thyroxine).

^a^Adjusted for age, ethnicity, smoking, socioeconomic status, and sex.

^b^CRP data were log transformed and are thus ratios.

^c^Using the 5 International Diabetes Federation (2006) criteria: central obesity, raised blood pressure, raised fasting plasma glucose, raised triglycerides, and reduced HDL cholesterol.

^d^Reference group sleep duration, 7.26 ± 0.26 hours (n = 195).

**Fig 1:**
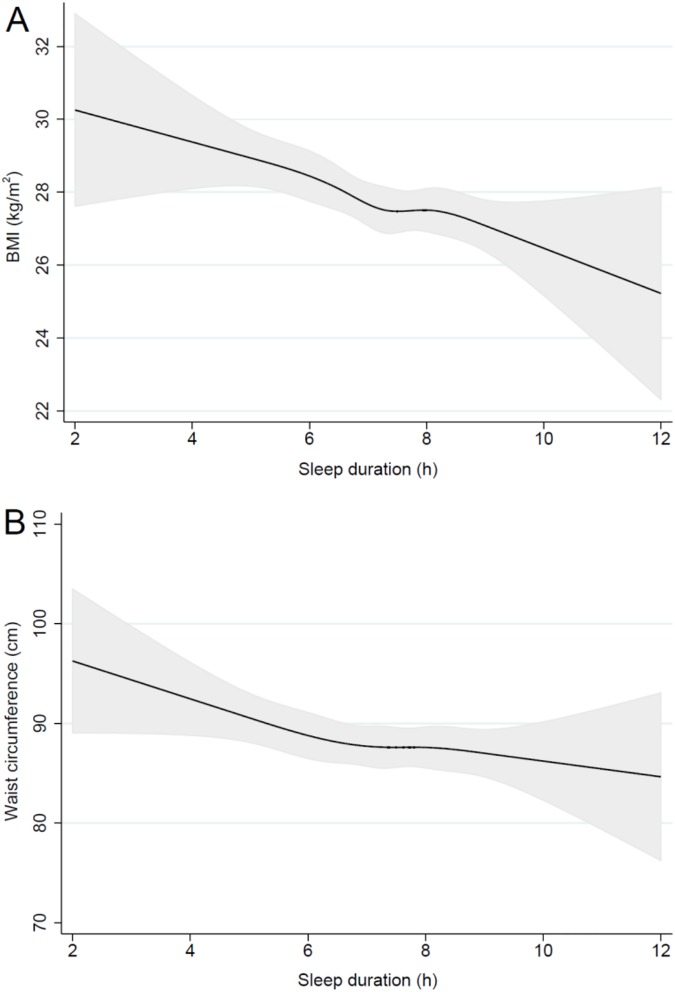
Sleep duration, BMI and waist circumference. Black lines plot the predicted BMI (A) and waist circumference (B) values with 95% confidence intervals (grey fill) for typical females from the sample (white, never smokers, lower managerial and professional occupation, using the mean age). Very similar associations were apparent in males.

Sleep duration was negatively associated with BMI and waist circumference, such that participants had 0.46 kg/m^2^ lower BMI values (95% CI -0.69 to -0.24 kg/m^2^, p < 0.001, Fig 1A) and 0.9 cm lower waist circumferences (95% CI -1.5 to -0.3 cm, p = 0.004, Fig 1B) per additional hour of sleep. Restricted cubic spline modelling showed that the negative association between sleep duration and these outcomes was linear.

After adjustment, sleep duration tended to be negatively associated with HbA1c and CRP, and positively associated with free T4, although these findings were not significant (p = 0.09 to 0.10). Following inclusion of BMI as a further adjustment, sleep duration was still not associated with any of these outcomes (P ≥ 0.16, Table 4).

**Table 4:** Sleep duration and measures of metabolic health, including body mass index as an adjustment.

Characteristic	Coefficient per additional hour of sleep (95% CI)	P value	n
Waist circumference (cm)	0.1 (-0.2 to 0.4)	0.56	1,157
HbA1c (%)	-0.02 (-0.06 to 0.01)	0.18	762
Fasting glucose (mmol/L)	-0.01 (-0.08 to 0.06)	0.77	726
CRP^a^	-0.01 (-0.13 to 0.10)	0.85	775
Triglycerides	-0.03 (-0.08 to 0.03)	0.38	772
LDL cholesterol	0.02 (-0.04 to 0.07)	0.54	758
HDL cholesterol	0.01 (-0.01 to 0.04)	0.22	774
Free T3 (pmol/L)	-0.01 (-0.07 to 0.04)	0.66	272
Free T4 (pmol/L)	0.16 (-0.06 to 0.37)	0.16	272
TSH (mIU/L)	0.02 (-0.19 to 0.23)	0.82	272

Adjusted for age, body mass index, ethnicity, smoking, socioeconomic status, and sex.

Legend: CI (confidence interval), CRP (C-reactive protein), HbA1c (glycated haemoglobin), HDL (high-density lipoprotein), LDL (low-density lipoprotein), OR (odds ratio), T3 (triiodothyronine), T4 (thyroxine).

^a^CRP data were log transformed and are thus ratios.

Of the five criteria used to diagnose an individual with metabolic syndrome, for each additional hour of sleep, participants had 0.10 fewer of these criteria in the unadjusted model (95% CI -0.20 to -0.02, p = 0.02), but this association was not significant following adjustment (p = 0.36). Similarly, for each additional hour of sleep, participants had 0.07 mmol/L lower triglyceride levels in the unadjusted model (95% CI -0.13 to -0.01, p = 0.02), but this association was not significant after adjustment (p = 0.11).

In linear regression analyses, sleep duration was not associated with fasting glucose, LDL cholesterol, free T3, or TSH. In the logistic regression analysis, sleep duration was not associated with the presence of metabolic syndrome (p ≥ 0.44).

## Discussion

Consistent with our predictions, sleep duration was negatively associated with BMI and waist circumference, and positively associated with HDL cholesterol levels. We also hypothesized that shorter sleepers would have impaired glucose metabolism and thyroid function, as well as higher systemic inflammation. Although not significant, sleep duration tended to be negatively associated with HbA1c, positively associated with free T4, and negatively associated with CRP. In unadjusted models, sleep duration also tended to be negatively associated with triglycerides and metabolic syndrome criteria, but these associations were no longer apparent after adjustment. In general, associations were strongly attenuated after inclusion of BMI as an adjustment, suggesting that higher BMI values contribute to metabolic dysfunction in shorter sleepers. Interestingly and contrary to our expectations sleep duration was not associated with diet. Collectively, these findings suggest that among UK adults longer sleepers have favorable metabolic profiles in comparison to shorter sleepers but not substantially different dietary habits.

Our observation that sleep duration was negatively associated with BMI and waist circumference is consistent with meta-analyses showing that short sleep is associated with obesity and central obesity [11, 12], and the magnitudes of the associations that we found are within the ranges of those documented previously. Interestingly, a recent study of a large group of UK adults showed that gene/environment interactions may be key determinants of obesity risk, as the adverse effects of various sleep behaviours (including short sleep) on adiposity were more pronounced among those with genetic predisposition to obesity [25]. These findings suggest a particular need to improve sleep patterns among people who are genetically susceptible to obesity.

Blood lipids are important determinants of risk of various metabolic diseases. HDL cholesterol helps offset excessive inflammation and regulates reverse cholesterol transport, thereby influencing risk of health problems such as atherosclerotic cardiovascular disease [26]. Whereas some studies have found that both short and long sleep may contribute to lower HDL cholesterol levels [27], we observed a more linear relationship. A consistency among studies, however, seems to be that short sleep is associated with adverse effects on lipid metabolism, including lower HDL cholesterol levels [28].

As is true of lipid metabolism, sleep restriction has detrimental effects on glucose metabolism [14], so we tested whether sleep duration was associated with fasting glucose and HbA1c, two markers used to diagnose type 2 diabetes. Sleep duration tended to be inversely associated with HbA1c but was not associated with fasting glucose. This discrepancy may be partly explained by the different factors that fasting glucose and HbA1c reflect [29]: HbA1c is a more stable marker of longer term glucose homeostasis, but fasting glucose is more susceptible to acute fluctuations resulting from variables such as diet and physical activity. In this way, we interpret our findings as providing tentative support for a role of chronic insufficient sleep in impaired long-term glucose homeostasis. A meta-analysis of prospective studies reported a U-shaped association between sleep duration and type 2 diabetes risk, but our restricted cubic spline models did not show nonlinear relationships with fasting glucose and HbA1c. This could reflect the relative scarcity of people whose sleep might be considered pathologically long. Whereas short sleepers comprised a significant proportion of the study population (9.9% reported sleeping less than 6 hours, for example), perhaps there were not enough long sleepers to see if long sleep might be pathological: Only 1.1% of participants reported sleeping longer than 10 hours.

Given our findings of greater adiposity, lower HDL cholesterol and a tendency to impaired glucose metabolism among shorter sleepers, we expected sleep duration to be associated with metabolic syndrome. Whereas the unadjusted linear regression model negatively associated sleep duration with number of metabolic syndrome criteria, however, this relationship was not apparent after adjustment. As the full complement of metabolic syndrome components was only available for 554 participants, we may have had insufficient statistical power to observe an association.

Thyroid hormones have myriad roles in metabolic regulation [30], so we determined if sleep duration was associated with thyroid function. Whereas experimental sleep restriction has been shown to acutely increase thyroid hormone secretion, our finding of borderline lower free T4 is more consistent with findings that chronic sleep restriction lowers free T4 levels [31]. As hypothyroidism predisposes people to weight gain, impaired thyroid function could be one mechanism by which long-term sleep loss increases susceptibility to obesity.

Higher CRP levels increase susceptibility to metabolic diseases such as type 2 diabetes [32]. Consequently, we also tested whether sleep duration was associated with blood CRP concentrations, finding that sleep tended to be negatively associated with CRP. This result is in line with evidence that sleep restriction generally induces a proinflammatory state, including elevated CRP levels [33], implying that dysregulated systemic inflammatory responses may be another means by which short sleep has adverse effects on metabolic regulation.

Epidemiologic studies have often associated short sleep with higher energy intake and have sometimes found that short sleep coincides with reduced dietary quality [34]. Some of the underlying mechanisms are well characterized [14], and meta-analyses of experimental sleep restriction studies have found that sleep restriction increases energy intake [35–37]. It is unclear why we did not find that short sleep was associated with increased energy intake and indices of processed food intake, especially when we found that sleep duration was inversely associated with BMI. Perhaps other components of energy balance were affected by sleep duration. It has been found, for example, that sleep restriction may acutely reduce resting metabolic rate [38] and could hence lead to a positive energy balance if energy intake remained constant. The extra energy cost of wakefulness could compensate for this, however, and a recent meta-analysis of sleep restriction intervention studies showed that curtailed sleep does not alter daily energy expenditure [37]. It could be that sleep duration has a reciprocal relationship with free-living physical activity, but we did not feel that there was sufficient experimental evidence that physical activity independently influences sleep duration to include physical activity as an adjustment.

Another possibility is that dietary underreporting is more pervasive among individuals with higher BMI and those wishing to lose fat mass [39], hindering our ability to observe a relationship between sleep duration and energy intake using such self-reported measures of diet. It is also possible that sleep duration may have been related to diet in a nonlinear manner. Based on our interpretation of the literature, however, we decided a priori to use linear regression analyses to model dietary outcomes. Finally, other sleep parameters such as sleep efficiency, sleep timing (a simple estimate of chronotype), and sleep timing variability may influence diet and metabolic health [40–42], but questions related these variables are not included in the NDNS-RP.

This study had several strengths, including comprehensive dietary assessment by way of 4 day estimated food diaries, thorough metabolic profiling, concurrent measures of diet and metabolic health, and the study of a representative adult population. Nevertheless, this work also had limitations. The study used self-reported sleep duration instead of a more objective measure such as actimetry or polysomnography, and participants were not asked about napping or effects of work schedules on sleep. Correlations between self-reported sleep duration and actimetry-estimated sleep duration of 0.43–0.45 have been reported in adults [43, 44], and self-reports tend to underestimate sleep duration, a discrepancy that may increase with longer self-reported sleep [43, 44]. Other limitations include the absence of information regarding behaviours that influence sleep (such as alarm use), missing data for some variables, and the possibility that participants’ behaviours were not rigorously standardized before collection of fasted samples.

As this was a cross-sectional study, it is of course not possible to infer that insufficient sleep results in adverse metabolic consequences. Prospective studies using more objective measures of sleep duration and quality are needed to better clarify the relationships between sleep, dietary habits and metabolic health. As recent studies have documented an array of beneficial effects of sleep extension on dietary choices and metabolic health in habitual short sleepers [45–47], the optimization of such interventions should be further studied.

In conclusion, longer sleepers generally had more favorable metabolic profiles, despite no associations between sleep duration and dietary intakes in this population. Our findings support the accumulating evidence showing an important contribution of short sleep to metabolic diseases such as obesity.
